# YY1 promotes pancreatic cancer cell proliferation by enhancing mitochondrial respiration

**DOI:** 10.1186/s12935-022-02712-w

**Published:** 2022-09-19

**Authors:** Bin Li, Junyi Wang, Jing Liao, Minghui Wu, Xiangshu Yuan, Hezhi Fang, Lijun Shen, Minghua Jiang

**Affiliations:** 1 Department of Laboratory Medicine, The Second Affiliated Hospital, Wenzhou Medical University, Wenzhou, 325027 Zhejiang China; 2 Key Laboratory of Laboratory Medicine, Ministry of Education; Zhejiang Provincial Key Laboratory of Medical Genetics; College of Laboratory Medicine and Life Sciences, Wenzhou Medical University, Wenzhou, 325035 China; 3 School of Laboratory Medicine and Life Sciences, Wenzhou Medical University, Wenzhou, 325035 China; 4 Department of Clinical Laboratory Examination, The Seventh Affiliated Hospital, Sun Yat-Sen University, Shenzhen, 518000 China

**Keywords:** *YY1*, PDAC, OXPHOS, Nucleotide metabolism, Aspartate

## Abstract

*KRAS*-driven metabolic reprogramming is a known peculiarity features of pancreatic ductal adenocarcinoma (PDAC) cells. However, the metabolic roles of other oncogenic genes, such as *YY1*, in PDAC development are still unclear. In this study, we observed significantly elevated expression of *YY1* in human PDAC tissues, which positively correlated with a poor disease progression. Furthermore, in vitro studies confirmed that *YY1* deletion inhibited PDAC cell proliferation and tumorigenicity*.* Moreover, *YY1* deletion led to impaired mitochondrial RNA expression, which further inhibited mitochondrial oxidative phosphorylation (OXPHOS) complex assembly and altered cellular nucleotide homeostasis. Mechanistically, the impairment of mitochondrial OXPHOS function reduced the generation of aspartate, an output of the tricarboxylic acid cycle (TCA), and resulted in the inhibition of cell proliferation owing to unavailability of aspartate-associated nucleotides. Conversely, exogenous supplementation with aspartate fully restored PDAC cell proliferation. Our findings suggest that *YY1* promotes PDAC cell proliferation by enhancing mitochondrial respiration and the TCA, which favors aspartate-associated nucleotide synthesis. Thus, targeting nucleotide biosynthesis is a promising strategy for PDAC treatment.

## Background

Pancreatic cancer is a type of gastrointestinal malignancy with an extremely poor prognosis [1, 2]. Its mortality rate is expected to surpass those of breast, prostate, and colorectal cancers by 2030, making it the second leading cause of cancer-related deaths [3]. Pancreatic ductal adenocarcinoma (PDAC) comprises approximately 90% of pancreatic cancer cases, with the majority of those patients carrying active *KRAS* mutations [4, 5]. The activation of tumor suppression genes, such as *CDKN2A/p16*, *TP53*, and *SMAD4*, also contributes to PDAC development [6, 7].

Generally, PDAC development involves metabolic remodeling to facilitate cancer cell proliferation. *KRAS* mutations can upregulate the expression of glycolytic pathway rate-limiting genes, such as phosphofructokinase-1, lactate dehydrogenase A, and hexokinase 2, consequently promoting PDAC tumorigenesis [8, 9]. Additionally, *KRAS* regulates the expression of hormone-sensitive lipase, to control the storage and utilization of lipid droplets, to fuel the invasive migration of PDAC cells [10]. CD9^high^, a subtype PDAC tumor-initiating cell, can enhance organoid formation by upregulating the expression of the neutral amino acid transporter B (*ASCT2*), located in the cell membrane, to enhance glutamine uptake [11]. Furthermore, the rapid development of PDAC is inseparable from nucleotide metabolism. *KRAS* promotes the expression of ribose-5-phosphate isomerase to accelerate nucleotide biosynthesis [8]. However, the regulation of nucleotide metabolism in PDAC is still unclear and needs elucidation.

Yin-Yang 1 (*YY1*), composed of 414 amino acids, belongs to GLI-Krüppel zinc finger protein family [12]. As a nuclear transcription factor, it contributes to the regulation of various cellular processes, such as autophagy, cell division, survival, and differentiation [13–15]. *YY1* has a dual function; it exerts tumor-promoting as well as -suppressive effects, depending on the cancer type. In breast cancer, its overexpression inhibits the growth and tumorigenesis of cancerous cells [16]. Conversely, its overexpression is associated with the proliferation of liver, prostate, gastric, colorectal, and ovarian cancer cells [17–20]. Therefore, *YY1* has different roles in various cancers, and its role in PDAC is still unclear.

Despite its tumor-promoting role, *YY1* contributes to the reprogramming of tumor cell metabolism, to aid the cell’s adaption to different microenvironments [21]. Particularly, it activates glucose-6-phospate dehydrogenase (*G6PD*) transcription, upregulates the activity of the pentose phosphate pathway (PPP), enhances nucleotide synthesis, and promotes cellular antioxidant defense by supplying nicotinamide adenine dinucleotide (NADH) to support tumor cell proliferation and tumorigenesis [22, 23]. Further, it regulates mitochondrial oxidative phosphorylation (OXPHOS)-related gene expression in the *PGC1* assistant [24]. However, the mechanism by which it regulates OXPHOS gene expression, to support nucleotide synthesis, needs to be clarified.

Therefore, we investigated the role of *YY1* in PDAC proliferation. Our results indicated that *YY1* is positively associated with PDAC development, while its knockdown (KD) inhibited PDAC cell proliferation. Our results are supported by biochemical and metabolic studies that revealed PDAC cell proliferation is promoted by *YY1*, which enhances nucleotide availability in a mitochondrial OXPHOS-dependent manner.

## Methods

### Cell lines and cell culture

The human pancreatic cancer cells PANC1, Pa-Tu-8988, BXPC-3, HEK293T, CFPAC, and MIA-PaCa2 were purchased from the Cell Bank of the Chinese Academy of Science (Shanghai, China), and the human pancreatic ductal epithelial hTERT-HPNE cell line (HPNE) was obtained from BaiRong Biotechnology (Shanghai, China). All the cell lines, authenticated via a short tandem repeat profiling analysis using Genetic Testing Biotechnology (Suzhou, China), were cultured in Dulbecco’s modified Eagle’s medium (DMEM) (Sigma-Aldrich, St. Louis, MO, USA) supplemented with 10% fetal bovine serum (FBS) (Sigma-Aldrich), 100 U/ml penicillin (Beyotime, Shanghai, China), 0.1 mg/ml streptomycin (Beyotime). All the cell lines were incubated at 37 °C in a 5% CO_2_ atmosphere.

### Generation of stable knockdown and transient knockdown cells

A stable KD cell model was generated using second-generation lentiviruses [25]. Lentiviral particles were produced via the co-transfection of the packaging psPAX2, envelope pMD2.G, and KD pLKO.1 vectors (1.25, 0.625 and 0.625 µg, respectively), that used Lipofectamine 3000 (Thermo Fisher Scientific, Cleveland, OH, USA) to carry shRNA sequences into 3 × 10^5^ HEK293T cells that were cultured in a 6-well dish. The *YY1* shRNA sequences were as follows: 5′-GACGACGACTACATTGAACAA-3′ and 5′-GCCTCTCCTTTGTATATTATT-3′. We used wild-type pLKO.1 plasmid as a control. We used the limiting dilution method, with puromycin (3 µg/ml), to select *YY1*-stable KD and control cell lines [26]. The pyruvate carboxylase (*PC*) transient KD cell line was generated using small interfering RNA (siRNA) provided by Ribobio Company (Guangzhou, China) (siRNA: F 5′-GACGGCGAGGAGATAGTGT-3′, R 5′-TGGCAATCTCACCTCTGTTGG-3′) and transfected control-siRNA (siN0000001-1-1, Ribobio). In brief, the siRNA was transfected into cells using the Lipofectamine™ RNAi MAX Transfection Reagent (Thermo Fisher Scientific), following the manufacturer’s protocol (Protocol Pub. No. MAN0007825 Rev.1.0, Thermo Fisher Scientific). A *PC* and *YY1* double KD cell line (YY1 KD siPC) was generated.

### Proliferation rates and colony formation

To perform the proliferation assay, 1 × 10^4^ cells were plated in each well of a 12-well dish (Corning). Thereafter, the cells were cultured in nutrient-restricted conditions, with 10% dialyzed FBS (Sigma-Aldrich) supplement, in DMEM (without pyruvate) (Sigma-Aldrich). After 12 h, the cells in each well were counted to determine the initial cell number. Furthermore, the cells with or without aspartate (20 mM) treatment were counted at 24 h intervals for up to 96 h. Thereafter, the proliferation rate was calculated. To perform a colony formation assay, we seeded 1 × 10^3^ cells in each well of a 6-well dish. When visible cell clones appeared, we fixed the cells with methanol for 15 min, after which they were stained with crystal violet (Beyotime) for 10 min. Finally, we used the ImageJ software to count the colonies [27].

### ATP measurement

For ATP measurement, 1 × 10^5^ cells were seeded in each well of 6-well dish (Corning) and the ATP level was measured using an ATP Bioluminescent Assay Kit (Sigma-Aldrich). ATP measurement was performed according to the protocol provide by manufacturer. To measure mitochondria-generated ATP, the cells were cultured with pyruvate and 2-DG (5 mM each) for 2 h. Furthermore, to determine the levels of glycolysis-generated ATP, the cells were cultured with 5 mM glucose and 1.25 µg/ml oligomycin, for 2 h.

### Oxygen Consumption Rate Assay

The oxygen consumption rate (OCR) assays were performed, as described previously [28], using the Oxygraph-2 k kit (OroborOSX, Innsbruck, Austria). After the cells were added to the chamber, we determined the basal OCR level. To this end, we added 2.5 mM oligomycin (Sigma-Aldrich) to the chamber to determine the uncoupling OCR. Finally, to determine the maximum OCR, we added cyanide 4-(trifluoromethoxy) phenylhydrazone (FCCP, 5 mM, Sigma-Aldrich).

### Apoptosis analysis

An Apoptosis Detection Kit (Keygen Biotech, Jiangsu, China) was used for apoptosis analysis. We collected 1 × 10^6^ single cells were collected, which were washed twice with cold PBS. The cell pellet was resuspended 500 µl binding buffer. Then, add 5 µl annexin V-EGFP and 5 µl propidium iodide (PI) to the tube and incubate at 23 °C for 15 min in the dark. Finally, cell fluorescence was measured using a NovoCyte flow cytometer (Agilent, Santa Clara, CA, USA).

### Cell cycle analysis

Cell cycle analysis was performed with the Cell Cycle Detection Kit (Keygen Biotech), 1 × 10^6^ single cells were collected, wash once with PBS, and resuspended the cell pellet with 500 µl 70% cold ethanol for 2 h at 4 °C. Thereafter, cells were washed twice with cold PBS before staining, and 500 µl PI/ RNaseA mixture was added to the tube and incubated in the dark for 30 min at 4 °C. In the next step, cells were then filtered for flow cytometry analysis. Finally, DNA content was determined using a NovoCyte flow cytometer and analyzed using the NovoCyte flow cytometer software (NovoExpress 1.5.0).

### Immunohistochemical analysis

Pancreatic tissue samples were collected from the Zhejiang Provincial People’s Hospital, including eleven normal pancreatic tissue samples and seventy-one pancreatic cancer tissue samples. Thereafter, immunohistochemical (IHC) analysis of tissue microarray (TMA) was performed as previously described [26]. Briefly, a targeted area of the tissues was removed from the paraffin-embedded tissue to obtain a TMA sample, which was then arrayed on a slide. This was followed by the deparaffinization and hydration of the samples, wash twice with PBS, then blocked endogenous peroxidase activity with 0.3% H_2_O_2_ for 15 min at 23 °C, wash three times with PBS, and then heat-induced epitope retrieval was performed. Afterwards, TMA samples were incubated with anti-YY1 (1:400, Proteintech, Wuhan, China) for 30 min at 23 °C, washed three times with PBS and incubate with fresh diaminobenzidine (DAB) for 5 min, then hematoxylin stain. Optical density (average OD value, AOD) of stained area were quantified using Image-Pro Plus software version 6.0 (Media Cybernetics, Rockville, MD, USA) and *YY1* expression level was analyzed according to AOD value.

### Immunoblotting

For sodium dodecyl sulfate polyacrylamide gel electrophoresis (SDS-PAGE) procedure, the cells were lysed with RIPA buffer (Cell Signaling Technology, Danvers, MA, USA), supplemented with 1 mM phenylmethylsulfonyl fluoride (Sangon Biotech, Shanghai, China), and incubated on ice for 15 min, and then centrifuged at 14,000*g* for 10 min at 4 °C, the supernatants were transferred into new tube, protein sample was boiled for 5 min. For blue native polyacrylamide gel electrophoresis (BNG), samples were lysed with 2.5% digitonin (w/v, Sigma-Aldrich), supplemented 1 mM PMSF (Sangon Biotech) and incubated on ice for 25 min, afterwards, centrifuged at 20,000*g* for 10 min at 4 °C, the supernatants were transferred into new tube. The proteins separated via BNG, or SDS-PAGE were transferred onto 0.22 µm polyvinylidene difluoride membranes (Bio-Rad, Hercules, CA, USA). Next, the membranes were blocked with 5% BSA (Sigma-Aldrich) for 1 h, and then incubated with the primary antibodies: anti-YY1 (66,281–1-Ig; 1:2000; Proteintech), anti-β-actin (sc-47778; 1:5000; Santa Cruz Biotechnology), anti-TOM70 (ab251925 1:10,000; Abcam), anti-ATP synthase subunit alpha (ab14748; 1:1000; Abcam), anti-COXI (MS404; 1:1000; Abcam), Abcam), anti-core2 (MS304; 1:1000; Abcam), anti-SDHA (ab14715; 1:1000; Abcam), and anti-GRIM19 (ab110240; 1:1000; Abcam, Cambridge, MA, USA), at 4 °C for 24 h. Thereafter, the membranes were incubated with a horseradish peroxidase-conjugated anti-rabbit/mouse IgG (#7074 / #7076; 1:2000; Cell Signaling Technology) secondary antibody for 4 h at 23 °C, and signal detection were performed with a Immun-Star HRP kit (Bio-Rad). Finally, the integrated optical density value (IOD) was quantified by Gel-Pro Analyzer version 4.0 (Media Cybernetics) and YY1 expression level was determined according to IOD.

### Metabolite profiling

To perform metabolite profiling experiments, samples were collected following the protocol provided by Metabo-Profile Biotechnology (Shanghai, China). Sample preparation were prepared according to a previously published method [29]. Briefly, MIA-PaCa2 and *YY1* KD cells (1 × 10^7^ per sample) were collected and washed twice with cold PBS. Thereafter, 1 mL of extraction solution buffer (methanol:acetonitrile:water = 2:2:1 (v/v)) was added to the sample. Then samples were then frozen in liquid nitrogen for 1 min, thawed, and vortexed for 30 s. The above-mentioned procedure was repeated, and thereafter, the samples were sonicated in an ice-water bath for 10 min, incubated at − 40 °C for 1 h, and then centrifuged at 12,000 rpm for 15 min at 4 °C. Finally, the supernatants were transferred into new glass vials, and sent to Metabo-Profile Biotechnology for metabolite measurements.

### Transcriptome profiling

For transcriptome profiling, samples were pre-treated following the protocol provided by the Novogene Corporation (Tianjin, China). In brief, MIA-PaCa2 and *YY1* KD cells (5 × 10^6^ per sample) were collected and washed with cold PBS, and total RNA extraction were performed with a RNeasy Mini Extraction Kit (Qiagen Sciences, Germantown, MD, USA), and mRNA were purified using Poly T-attached magnetic beads. To perform reverse transcription using random hexamer primers, the M-MuLV system was used. Library construction as well as sequencing were carried out by Novogene Corporation (Tianjin, China) using a HiSeq 2000 platform (Illumina, San Diego, CA, USA). In the control group, one replicate showed a large deviation from the other two; thus, we used the two-versus-two comparison method for further analysis. The metabolism gene list was obtained from a previously published study [30].

### Mitochondrial RNA, YY1, and PC transcription analysis

Mitochondrial DNA transcripts were measured via quantitative polymerase chain reaction (qPCR) using a Quantagene q225 system (Kubo Tech, Beijing, China). Total RNA was extracted using TRIzol reagent (Thermo Fisher Scientific) following the manufacturer’s protocol. Thereafter, 500 ng of the extracted RNA was analyzed using a reverse transcription kit (Takara Biotechnology, Dalian, China). Further, fluorogenic SYBR Green (Bio-Rad) was used for qPCR; the reaction conditions were as follows: 95 °C for 120 s, 95 °C for 10 s, and 60 °C for 30 s, and the amplification primer sequences were as shown in Table 1.

**Table 1:** Amplification primer sequences

Gene	Primer	Sequence
*YY1*	YY1-F YY1-R	5′-ACCTGGCATTGACCTCTCAG-3′ 5′-TGCAGCCTTTATGAGGGCAAG-3
*PC*	PC-F PC-R	5′-GACGGCGAGGAGATAGTGT-3′ 5′-TGGCAATCTCACCTCTGTTGG-3′
*β-Actin*	Actin-F Actin-R	5′-GACCTGTACGCCAACACAGT-3′ 5′-AGTACTTGCGCTCAGGAGGA-3′
*mtND1*	mtND1-F mtND1-R	5′-CCCATGGCCAACCTCCTACTCCTC-3′ 5′-AGCCCGTAGGGGCCTACAACG-3′
*mtND2*	mtND2-F mtND2-R	5′-AACCCTCGTTCCACAGAAGCT-3′ 5′-GGATTATGGATGCGGTTGCT-3′
*mtND3*	mtND3-F mtND3-R	5′-AAAATCCACCCCTTACGAGTG-3′ 5′-GTTTGTAGGGCTCATGGTAGG-3′
*mtND4(L)*	mtND4(L)-F mtND4(L)-R	5′-CCCACTCCCTCTTAGCCAATATT-3′ 5′-TAGGCCCACCGCTGCTT-3′
*mtND5*	mtND5-F mtND5-R	5′-CTACCTAAAACTCACAGCCCTC-3′ 5′-GGGTAGAATCCGAGTATGTTGG-3′
*mtND6*	mtND6-F mtND6-R	5′-GCCCCCGCACCAATAGGATCCTCCC-3′ 5′-CCTGAGGCATGGGGGTCAGGGGT-3′
*mtCO1*	mtCO1-F mtCO1-R	5′-GCCATAACCCAATACCAAACG-3′ 5′-TTGAGGTTGCGGTCTGTTAG-3′
*mtCO2*	mtCO2-F mtCO2-R	5′-ACCAGGCGACCTGCGACTCCT-3′ 5′-ACCCCCGGTCGTGTAGCGGT-3′
*mtCO3*	mtCO3-F mtCO3-R	5′-CCTTTTACCACTCCAGCCTAG-3′ 5′-CTCCTGATGCGAGTAATACGG-3′
*mtCytB*	mtCytB-F mtCytB-R	5′-CCCACCCTCACACGATTCTTTA-3′ 5′-TTGCTAGGGCTGCAATAATGAA-3′
*mtATP6*	mtATP6-F mtATP6-R	5′-TTATGAGCGGGCACAGTGATT-3′ 5′-GAAGTGGGCTAGGGCATTTTT-3′
*mtATP8*	mtATP8-F mtATP8-R	5′-CCCCATACTCCTTACACTATTCC-3′ 5′-CGTTCATTTTGGTTCTCAGGG-3′

### Analysis of public dataset

The gene expression levels of YY1 in pancreatic cancer were obtained from the website http://gepia.cancer-pku.cn. First select the Boxplot sub-option in Expression DIY, enter the YY1 to be queried, then the name of the cancer to be queried, find the PAAD and add it to the datasets, select the Match TCGA normal and GTEx data option, and the rest are set by default. The results are displayed in the form of plot. The relationship between YY1 expression and patient survival was queried through the same website. Select the survival option, select survival plots, enter the corresponding gene name YY1, select the type of cancer to be queried and select group cutoff. In this study, quartile was used for analysis, and the rest of the settings were based on the default values. Click on plot to generate a graph of the relationship between the expression level of YY1 in patients and prognosis survival.

### Statistical analysis

Data were presented as the mean ± SEM based on at least three independent replicate experiments. Significant differences were evaluated by performing independent Student's t-test or paired Student's t-test using SPSS software v21.0 (IBM, Armonk, NY, USA). The data were plotted using Prism 8.0 (GraphPad Software, San Diego, CA, USA) and statistical significance was set at P < 0.05. Significance level: *P < 0.05, **P < 0.01, ***P < 0.001.

## Results

### YY1 is elevated in PDAC and is positively correlated with a poor prognosis in patients with PDAC

**Fig. 1:**
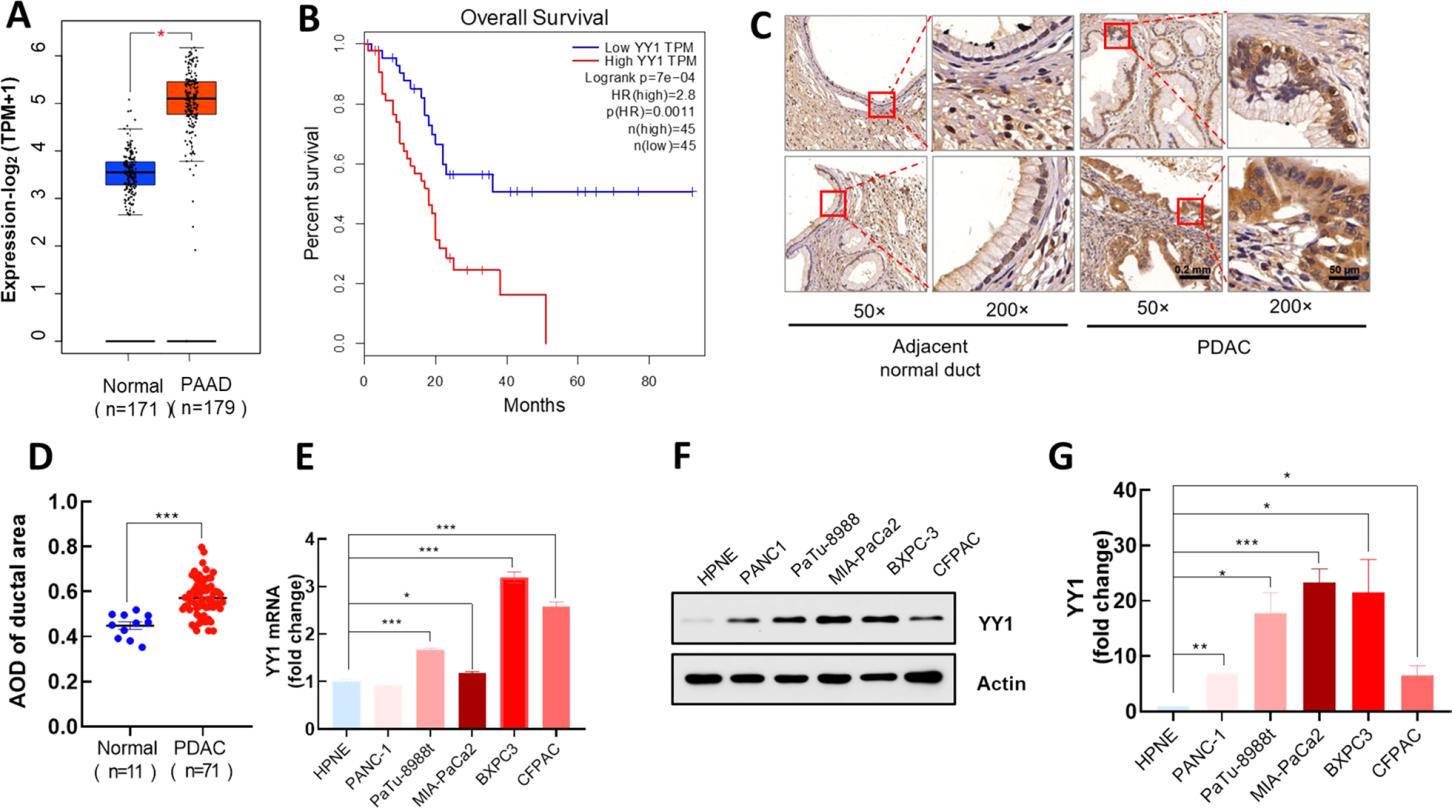
*YY1* is elevated in PDAC and is positively correlated with a poor prognosis in patients with PDAC. **A**, **B** *YY1* expression and survival plots in pancreatic adenocarcinoma (PAAD) provided by the GEPIA server (http://gepia.cancer-pku.cn). For the expression plot (**A**), the threshold was set as follows: log2-fold change < 1, P < 0.01, and for the survival plot (**B**), the high and low expression groups were split by inter-quartile ranges, and the hazards ratio (HR) was calculated based on the Cox PH model. **C** Immunohistochemical (IHC) analysis of TMA. TMAs were incubated with anti-*YY1* antibodies and stained with DAB and hematoxylin. *YY1* expression in adjacent normal (n = 11) and malignant pancreatic ducts (n = 71) is shown at different magnifications (50 × , scale bar = 200 μm; 200 × , scale bar = 50 μm). **D** Quantitative analysis of *YY1* expression in TMAs. Quantification of *YY1* expression represented as average optical density (AOD). **E** Relative *YY1* mRNA levels in PDAC and HPNE cell lines. **F** *YY1* protein levels in PDAC and HPNE cell lines. **G** Statistical analysis of *YY1* western blotting results (n = 3)

Upon comparing data from The Cancer Genome Atlas (TCGA) and Genotype-Tissue Expression (GTEx) databases, the expression level of *YY1* was found to be significantly higher in cancerous than in healthy pancreatic tissues (Fig. 1A). Furthermore, survival analysis indicated that a high *YY1* expression was associated with a poor PDAC prognosis (Fig. 1B). Additionally, IHC analysis also revealed a significant increase in *YY1* expression in the cancerous pancreatic ductal area (Fig. 1C, D). Quantification of *YY1* mRNA and protein expression levels in PDAC and HPNE cells revealed that *YY1* was significantly upregulated in PDAC cells (Fig. 1E–G). Although PANC1 and HPNE showed no significant difference with respect to *YY1* expression at the mRNA level (Fig. 1E), western blotting showed that *YY1* expression was significantly elevated in PANC1 cells (Fig. 1G).

### Knockdown of YY1 inhibits PDAC cell proliferation

**Fig. 2:**
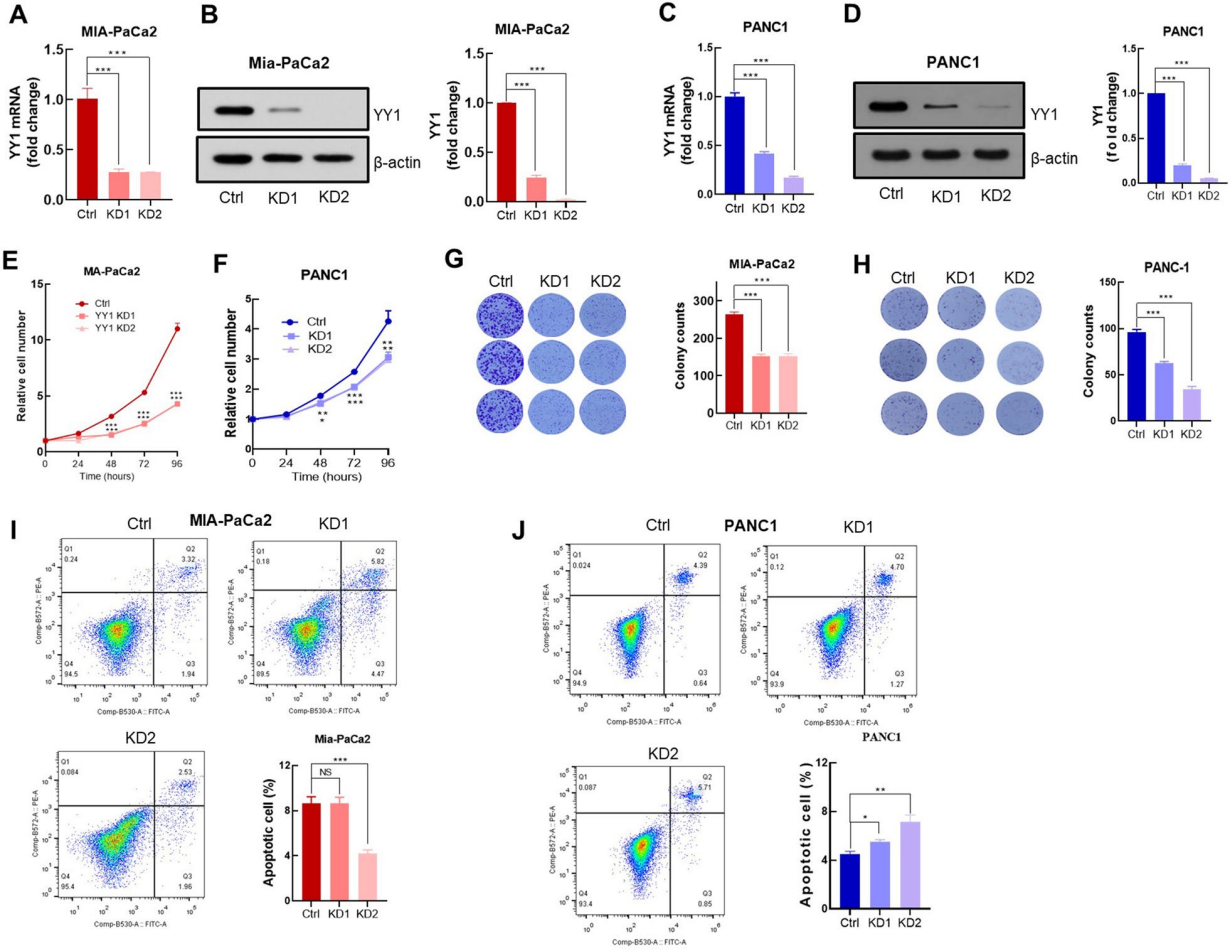
*YY1* knockdown inhibits PDAC cells proliferation. **A** Relative *YY1* mRNA levels in MIA-PaCa2 control and *YY1* KD cells (n = 3 per group) (Western blot analysis of *YY1* expression in MIA-PaCa2 and *YY1* KD cells; statistical analysis was based on *YY1* levels corresponding to the MIA-PaCa2 control and *YY1* KD cells (n = 3 per group). **C** Relative *YY1* mRNA transcription levels in PANC1 control and *YY1* KD cells (n = 3 per group). **D** *YY1* levels in MIA-PaCa2 control and *YY1* KD cells detected via western blotting; statistical analysis was based on *YY1* levels corresponding to the MIA-PaCa2 control and *YY1* KD cells (n = 3 per group). **E** Proliferation rate of the MIA-PaCa2 control and *YY1* KD cells; data were normalized to cell number at t = 0. **F** Proliferation rate of PANC1 control and *YY1* KD cells determined as in **E**. **G** (left) Colony formation in crystal violet-stained MIA-PaCa2 control and *YY1* KD cells after 10 days of cell culturing, (right) Colony number counted using the ImageJ software. **H** Colony formation in PANC1 control and *YY1* KD cells determined as in **G**. **I** Apoptosis of MIA-PaCa2 control and *YY1* KD cells measured via flow cytometry (n = 3 per group). The bar graph located to the lower right depicts the statistical analysis of cell apoptosis. **J** Cell apoptosis of PANC1 control and *YY1* KD cells determined as in **I**

To determine the role of *YY1* in PDAC, an in vitro* YY1* KD cell model was established in MIA-PaCa2 and PANC1 cell lines. The *YY1* KD ratios were determined at both mRNA and protein levels (Fig. 2A–D). Loss-of-function experiments were used to explore the effects of *YY1* KD on PDAC cell proliferation and colony formation capacity. Compared with those of the control cells, the proliferation rate (Fig. 2E, F) and clone formation ability (Fig. 2G, H) of *YY1* KD cells were significantly reduced. To confirm that this reduction was not due to *YY1* KD-induced apoptosis, we performed apoptosis assays and flow cytometry using the classical ANNEXIN-V and PI double staining method. The apoptosis index was determined by calculating the ratio of the ANNEXIN-V^+^ population (Lower Right: viable apoptotic cell) and the ANNEXIN-V ^+^/PI^+^ population (Upper Right: non-viable apoptotic cell) in the entire sample. Cell apoptosis analysis demonstrated that cell proliferation arrest, induced by *YY1* KD, was not a result of apoptosis (Fig. 2I, J).

### Knockdown of YY1 impairs cellular nucleotide homeostasis

**Fig. 3:**
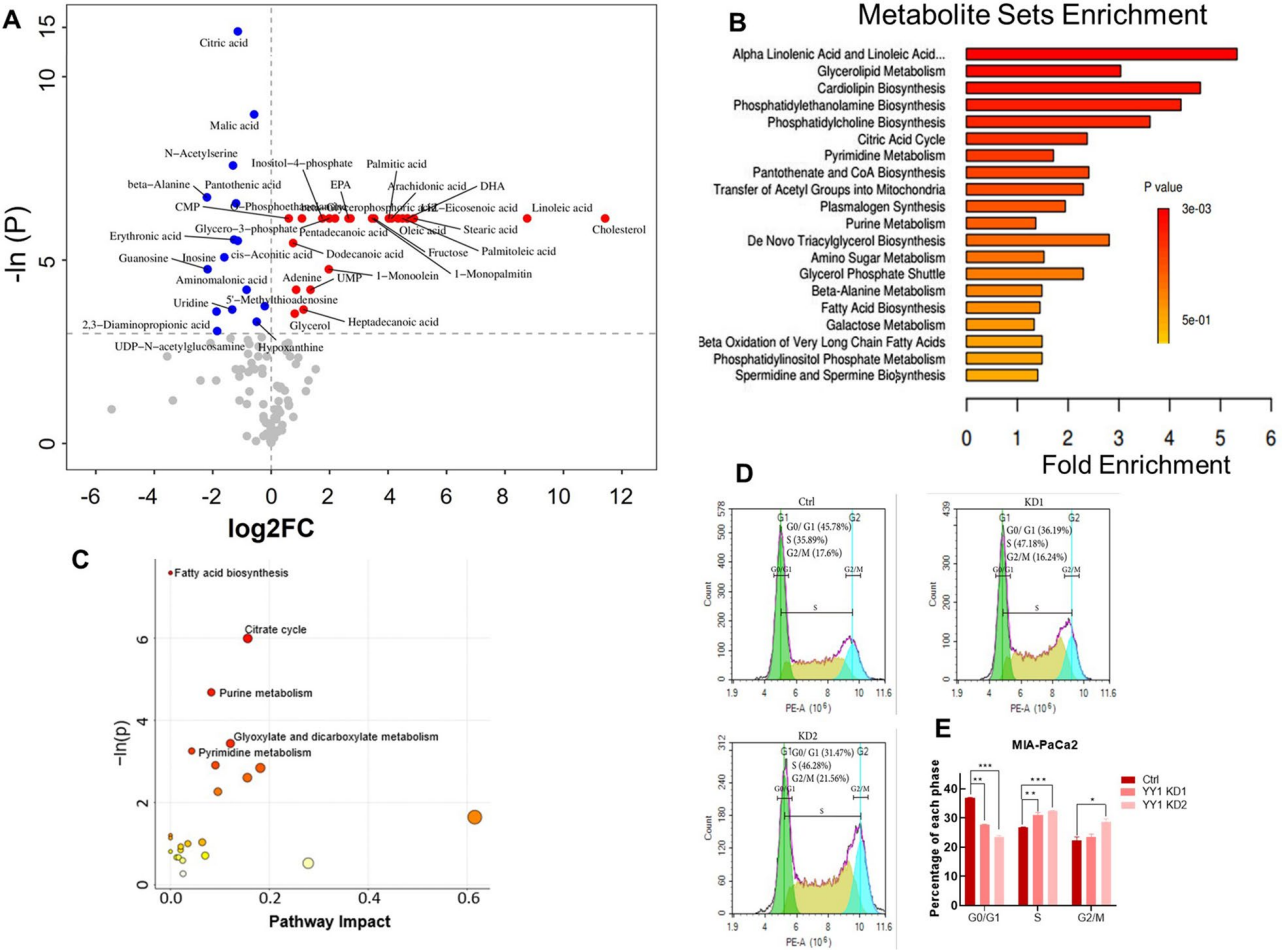
Knockdown of *YY1* impairs cellular nucleotide homeostasis. **A** Metabolite profiling results corresponding to MIA-PaCa2 control and *YY1* KD cells. The red and blue points represent up- and down-regulated YY1 KD cell metabolites, respectively (P < 0.05, n = 6 per group). **B** Metabolite Sets Enrichment analysis of whole metabolites. **C** Pathway impact analysis of whole metabolites, pathways with P < 0.05 were marked in the figure. **D** DNA content in MIA-PaCa2 control and *YY1* KD cells (n = 3 per group). **E** Statistical analysis of the cell cycle

Cell proliferation requires a large number of substrates and an adequate energy supply. Metabolite profiling of MIA-PaCa2 *YY1* KD and control cells showed that upon removal of *YY1*, 24 metabolites were significantly up-regulated and 15 were significantly down-regulated upon removal of *YY1* (Fig. 3A). Furthermore, Metabolite Sets Enrichment analysis showed that the metabolites involved in citrate cycle, pyrimidine metabolism, and purine metabolism were significantly enriched in *YY1* KD cells compared to those in control cells (Fig. 3B). A metabolite pathway impact analysis also indicated that fatty acid biosynthesis, citrate cycle, pyrimidine metabolism, purine metabolism were enriched in *YY1* KD cells (Fig. 3C). Cell cycle analysis revealed that the proportion of cells in the S phase was significantly increased in *YY1* KD cells (Fig. 3D, E). These results indicate that *YY1* KD impairs nucleotide metabolism, the probable major cause of cell proliferation arrest [31, 32].

### YY1 knockdown downregulates mitochondrial genome-encoded OXPHOS gene expression

**Fig. 4:**
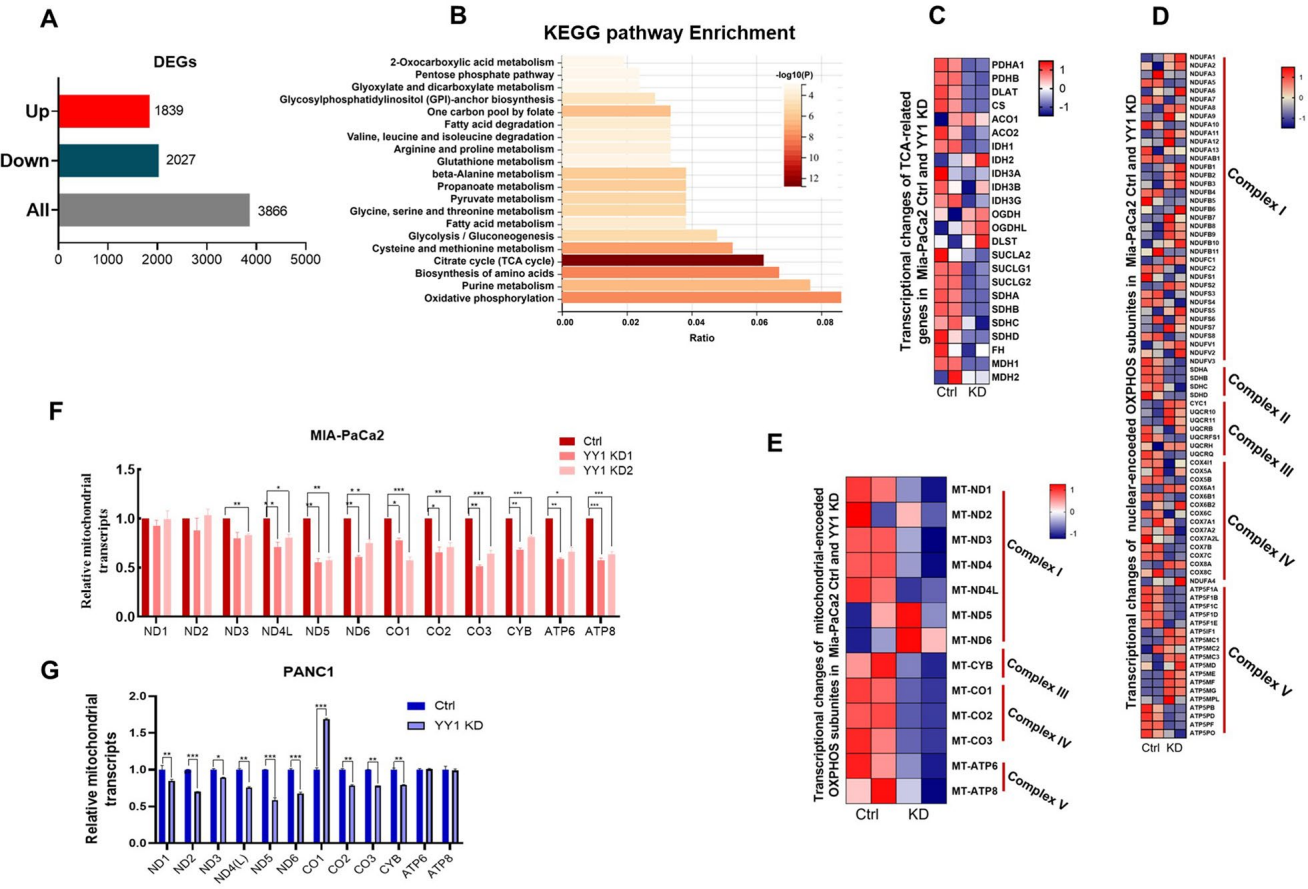
*YY1* knockdown downregulates mitochondrial genome-encoded OXPHOS gene expression. **A** Differentially expressed genes in MIA-PaCa2 control and *YY1* KD cells. The threshold was set as: P-adj < 0.05, |log2(Ctrl/*YY1* KD) |> 0. **B** KEGG pathway enrichment analysis of metabolic genes in *YY1* KD downregulated genes. The Gene Set Enrichment Analysis (GSEA) was performed based on the KEGG database (https://www.genome.jp/kegg/). The threshold was set as: log_2_(Ctrl/*YY1* KD) > 0. Heatmap representation of tricarboxylic acid cycle (TCA) gene expression (**C**), nuclear encoded OXPHOS genes expression (**D**), and mitochondrial genome encoded OXPHOS gene expression **E** in MIA-PaCa2 control and *YY1* KD cells as determined via RNA-seq. The data are from two independent experiments and the values are normalized via the Z-Score and color-coded as indicated (n = 2 per group). Relative mitochondrial-encoded OXPHOS protein expression in MIA-PaCa2 control and *YY1* KD cells (**F**) and PANC1 control and *YY1* KD cells (**G**), as determined by RT-qPCR. *β-*actin was the internal control (n = 3 per group)

*YY1* KD impairs nucleotide metabolism; however, the causative mechanism is still unclear. Furthermore, *YY1* is a nuclear transcription factor that regulates the transcription of numerous genes. A total of 3866 significantly differentially expressed genes were detected in MIA-PaCa2 *YY1* KD and control cells, with 1839 and 2027 genes upregulated and downregulated, respectively, in *YY1* KD cells (Fig. 4A). Furthermore, KEGG pathway cluster analysis of the downregulated genes in *YY1* KD cells revealed that the top 20 metabolism-related pathways included the oxidative phosphorylation pathway, purine metabolism, biosynthesis of amino acids, and the citrate cycle (TCA) (Fig. 4B). KEGG pathway analysis also revealed that *YY1* KD primarily affected the TCA cycle, OXPHOS, and purine metabolism pathway-related gene transcription. Specifically, TCA-related gene expression was generally reduced in *YY1* KD cells (Fig. 4C), while nuclear genome encoding OXPHOS genes did not show apparent variations between the *YY1* KD and control cells; however, mitochondrial genome-encoded OXPHOS genes showed reduced expression in *YY1* KD cells (Fig. 4D, E). This difference was confirmed via RT-qPCR analysis of MIA-PaCa2 and PANC1 YY1 KD cell lines (Fig. 4F, G).

### YY1 Knockdown impairs mitochondrial function

**Fig. 5:**
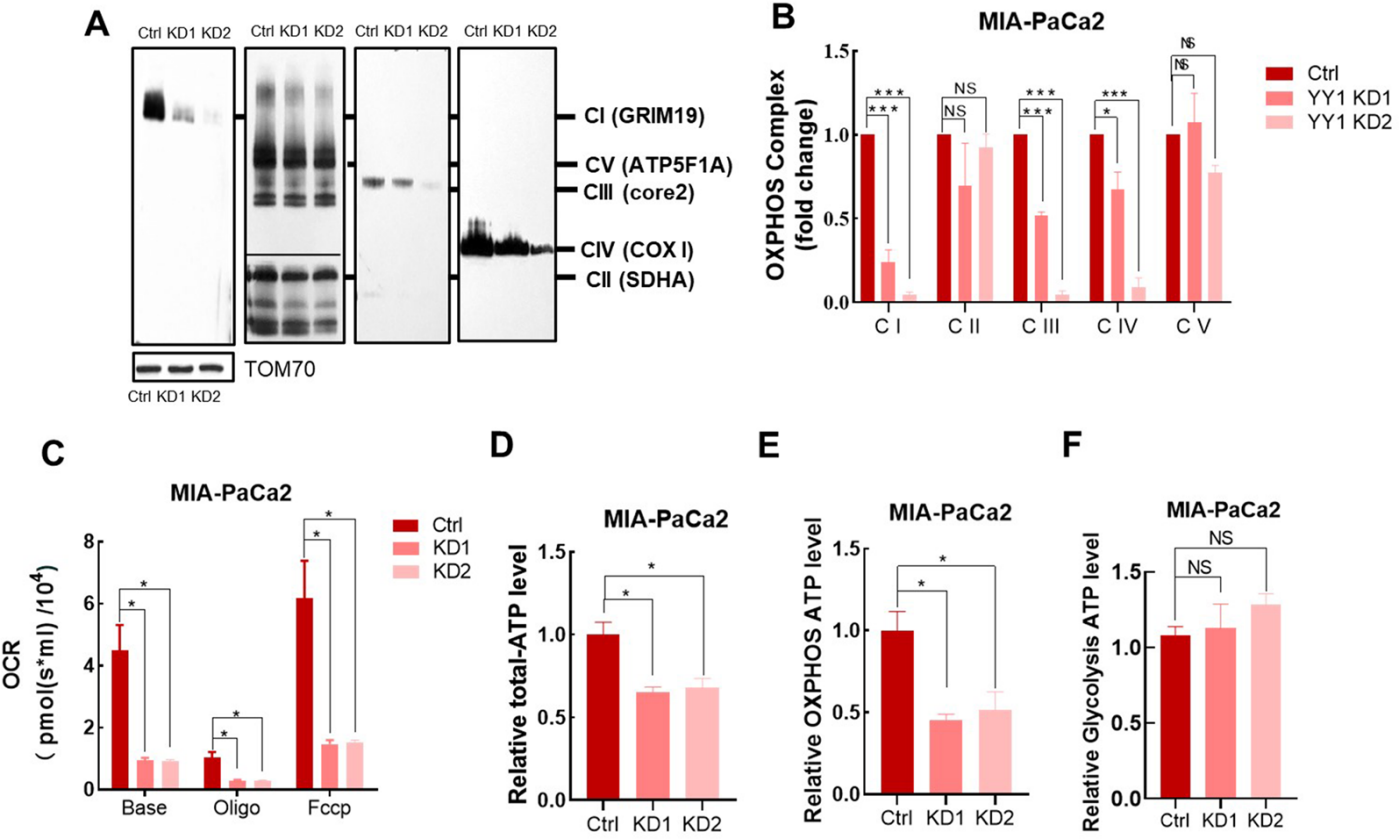
YY1 knockdown impairs mitochondrial function in pancreatic cancer cells. **A** Respiratory chain single complex expression levels were determined in MIA-PaCa2 control and *YY1* KD cells via BNG (CI: anti-GRIM19, CII: anti-SDHA, CIII: anti-core2, CIV: anti-COX I, CV: anti-ATP synthase subunit alpha (ATP5F1A), anti-TOM70 as loading control, n = 3 per group). **B** Quantification of single complex expression, based on density (n = 3 per group). **C** Oxygen consumption rate of the MIA-PaCa2 control and *YY1* KD cells. Relative ATP levels in MIA-PaCa2 control and *YY1* KD cells (n = 3 per group). Whole cell ATP level (**D**), cells were incubated with 5 mM 2-DG and 5 mM pyruvate for the measurement of mitochondria-generated ATP (**E**) and cells incubated with 5 mM glucose and 1.25 µg/mL oligomycin for the measurement of glycolysis-generated ATP (**F**) (n = 3 per group)

*YY1* KD mainly affected the expression of genes related to TCA and OXPHOS, which are closely associated with mitochondrial function. The downregulated expression of mitochondrial OXPHOS-related genes could alter OXPHOS complexes formation. BNG results showed that *YY1* KD downregulated the mitochondrial OXPHOS complex (Fig. 5A, B). Furthermore, OCR analysis demonstrated that *YY1* KD cells had significantly reduced basal and maximum OCRs (Fig. 5C). Our results also indicated that whole cell ATP production capacity, which depends on OXPHOS, was significantly reduced in *YY1* KD cells (Fig. 5D, E), while glycolysis-dependent ATP production was unaffected (Fig. 5F).

### Aspartate restores cell proliferation in YY1KD cells due to impaired mitochondrial respiration

**Fig. 6:**
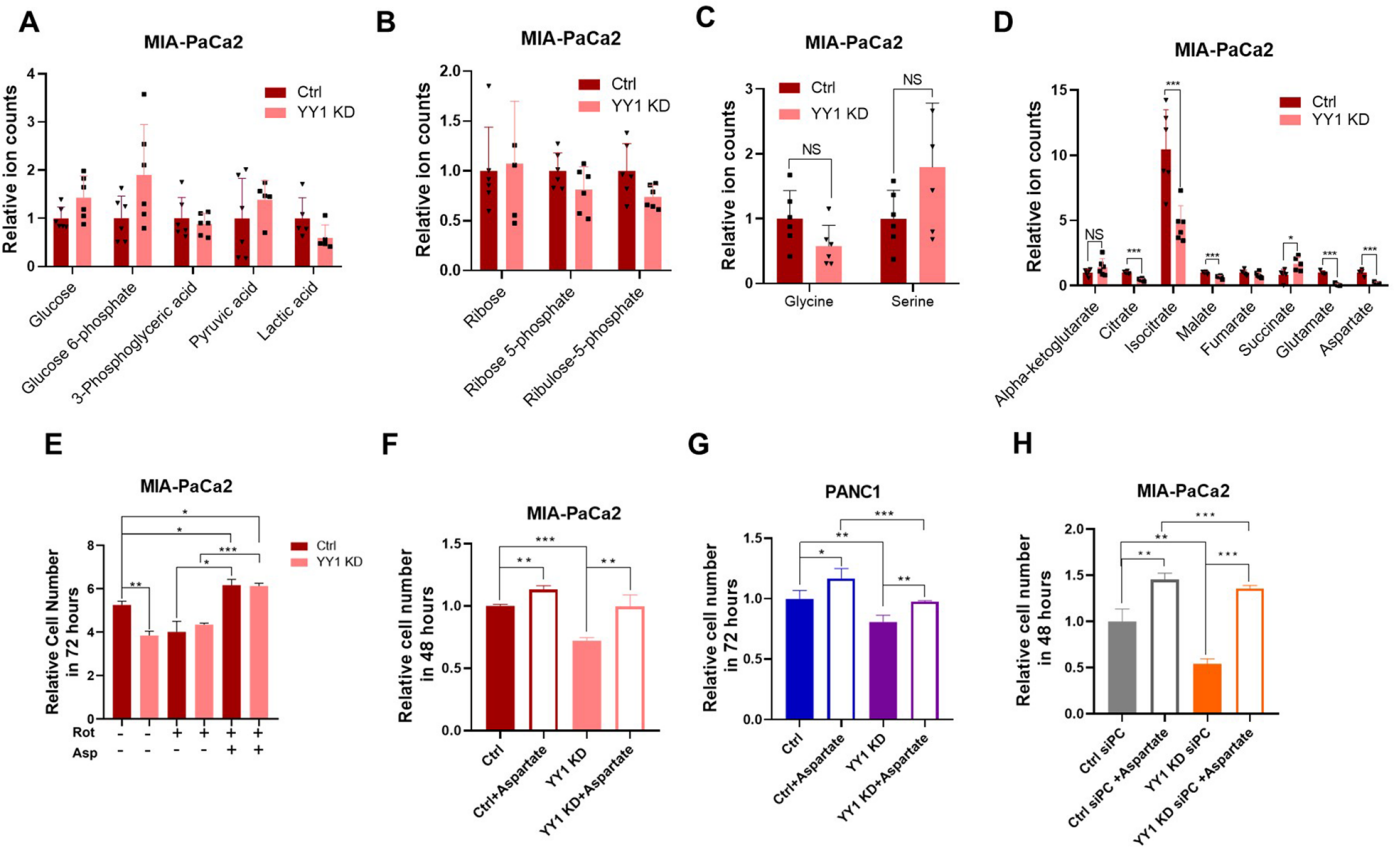
Aspartate restores cell proliferation in *YY1* KD cells due to impaired mitochondrial respiration. **A-D** Gas Chromatography/Mass Spectrometry (GC/MS) quantification of different metabolic pathway metabolites in MIA-PaCa2 control and *YY1* KD cells. **A** Glycolysis, **B** pentose phosphate pathway, **C** one carbon, and **D** TCA (n = 3 per group). **E** Proliferation rates of MIA-PaCa2 control and *YY1* KD cells treated with mitochondrial OXPHOS inhibiter, rotenone (25 nM), with or without aspartate (20 mM). Cell number was normalized with t = 0 (n = 3 per group). **F** Proliferation rates of MIA-PaCa2 control and *YY1* KD cells were calculated with or without aspartate (20 mM). Cell number was normalized with t = 0 (n = 3 per group). **G** Proliferation rates of PANC1 control and *YY1* KD cells determined as in **F** (n = 3 per group). **H** Proliferation rates of MIA-PaCa2 *PC* KD cells determined in the presence or absence of aspartate (20 mM). Cell number was normalized with t = 0 (n = 3 per group)

Given that *YY1* KD induced cell cycle arrest and impaired mitochondrial function, the relationship between nucleotide metabolism and mitochondrial function was investigated. Our results in this regard indicated that nucleotide biosynthesis depends on multiple metabolic pathways, such as glycolysis, PPP, the TCA cycle, and one-carbon metabolism. Metabolomics results also showed no significant difference between *YY1* KD and control cells with respect to the glycolysis pathway, PPP, and one-carbon cycle (Fig. 6A–C); however, TCA-related metabolites were significantly reduced in *YY1* KD cells (Fig. 6D). Further, the levels of glutamate and aspartate were drastically decreased in *YY1* KD cells. Aspartate provides a carbon backbone for de novo pyrimidines. To determine whether mitochondrial function deficiency inhibited aspartate biosynthesis-induced proliferation, we treated *YY1* KD and control cells with the OXPHOS inhibitor, rotenone (CI), which eliminates the proliferation advantage of control the cells. The addition of supra-physiological concentrations of aspartate into the culture medium restored the proliferation arrest caused by OXPHOS inhibitors (Fig. 6E). Thus, it appeared that the reason for *YY1* KD inhibiting cell proliferation was the inability to produce sufficient aspartate for nucleotide synthesis. After aspartate over-supplementation in the culture medium, *YY1* KD cell proliferation returned to normal; however, it was still lower than that corresponding to the control cells with aspartate (Fig. 6F). Additionally, aspartate supplementation reversed YY1 KD-induced cell proliferation arrest in the PANC1 cell line (Fig. 6G). Reportedly, in mammalian cells, de novo aspartate synthesis occurs in the mitochondria, where glutamic-oxaloacetic transaminase 2 (GOT2) catalyzes the transamination of glutamate to oxaloacetate (OAA), from aspartate and alpha-ketoglutarate [33, 34]. In the culture medium, glutamine is typically over-supplemented, while OAA, a non-essential nutrient, can be produced through a TCA cycle-dependent and -independent pathway, in which *PC* can convert pyruvate to OAA [35]. MIA-PaCa2 *YY1* and *PC* double-KD cells were unable to proliferate, but their proliferation could be restored with the addition of aspartate (Fig. 6H). These results suggested that the proliferation rate difference in between *YY1* KD and control cells was caused by TCA-dependent aspartate synthesis.

## Discussion

Pancreatic cancer is a disease that involves multiple gene pathways, and approximately 90% of pancreatic cancers contain *KRAS*^G12D^. Additionally, the inactivation of *P53* further accelerates pancreatic cancer development [36]. To adapt to the hypo-vascular nature of pancreatic cancer, which is usually characterized by oxygen and nutrient deficiency, oncogenic *KRAS* promotes glucose transporter (*GLUT1*) and hexokinase gene transcription to enhance glucose transport and utilization [8]. Moreover, pancreatic cancer cells can obtain nutrients through various means, be it *KRAS-* or *P53*-dependent or -independent [37–41]. In this study, we observed an increase in *YY1* expression in PDAC cell lines, and associated with a poor prognosis. Previous reports revealed that *YY1* can downregulate pancreatic cancer development through the YY1-CDKN3-MDM2/P53-P21 axis [42]. However, contrary to previous research, CDKN3 showed no significant difference in the MIA-PaCa2 *YY1* KD cell line (data not shown), possibly caused by the genomic variance in PDAC cells [43]. Studies have shown that *KRAS* can activate *YY1* transcription through the NF-κB signaling pathway. The activated *YY1* downregulates the expression of the tumor suppressor gene miR-489, thereby promoting the migration and metastasis of pancreatic cancer cells [20]. In addition, we explored the function of *YY1* in pancreatic cancer using a series of loss-of-function assays. The results indicated that *YY1* KD inhibited cell proliferation, which could be reversed by aspartate supplementation. Further investigations demonstrated that *YY1* KD reduced mitochondrial OXPHOS gene transcription, leading to mitochondrial dysfunction.

The function of mitochondria can be summarized as follows: it (1) provides ATP for various cell activities, such as cell proliferation, protein transport, and migration; (2) produces substrates for the biosynthesis of macromolecules, such as proteins, lipids, and nucleotides and [23, 31]; (3) regulates cell apoptosis and signaling [44–46]. Normal cells transport pyruvate into the mitochondria for ATP production, while cancer cells, independent of the mitochondria, convert it into lactic acid for complete oxidation, even with sufficient oxygen (Warburg effect) [47]. Mitochondrial OXPHOS is primarily an ATP-producing, catabolic process in cells [48, 49]. However, glycolysis can also produce sufficient ATP to support cell survival [50]; in cancer cells, OXPHOS is usually defective [51, 52]. However, mitochondria still play a very important role in cancer cell proliferation [31, 53]. NADH, produced by glycolysis, is transported from the cytoplasm to the mitochondria to regenerate NAD^+^, which relies on the malate-aspartate shuttle [54]. Furthermore, the transport of aspartate from mitochondria to the cytoplasm relies on the malate-aspartate shuttle. The concentration of aspartate, which is mainly synthesized in the mitochondria via transamination catalyzed by *GOT2*, in human blood is extremely low (0–15 µM) [55]. Besides its role as an important component of proteins, aspartate provides a carbon backbone for nucleotide synthesis [56]. In this study, cell cycle analysis of *YY1* KD cells demonstrated that they arrested in the S phase, indicating that they were unable to synthesize sufficient nucleotides for cell proliferation. Additionally, the observed metabolic profiles indicated that the metabolic pathways involved in nucleotide synthesis, such as the glycolysis pathway, PPP, and one-carbon cycle pathway, were unaffected in *YY1* KD cells. After adding OXPHOS inhibitor to the culture medium of *YY1* KD and control cells, the proliferation advantage of the control cells disappeared, while proliferation arrest was reversed by aspartate. Thus, we inferred that the *YY1* KD cell cycle arrest was due to impaired aspartate biosynthesis.

When supra-physiological levels of aspartate were added to the *YY1* KD cell culture medium, the proliferation of *YY1* KD cells became normal, confirming that the difference in proliferation ability between *YY1* KD and control cells was caused by differences in intracellular aspartate concentration. Given that aspartate is formed from OAA, the OAA content of the mitochondria determines its biosynthesis [57]. When OAA is converted to aspartate, the TCA cycle slows down due to a lack of intermediates, which can be replenished using glutamate and pyruvate. The glutamate content in *YY1* KD cells was lower than that in control cells, and this possibly impeded aspartate synthesis in *YY1* KD cells via the TCA cycle. Another pathway by which OAA is replenished is the conversion of pyruvate to OAA by *PC*, which is independent of the TCA pathway [58, 59]. After *PC* knock down in *YY1* KD cells and control cells, the *YY1* KD cells could not proliferate; thus, cell death was observed. However, the control cells still showed the ability to proliferate. After adding aspartate to the *PC* KD cell culture medium, the difference between the *YY1* KD and control cells in terms of proliferation disappeared. This result can be explained by the fact that TCA-dependent aspartate synthesis was primarily responsible for the inhibition of *YY1* KD cell proliferation.

## Conclusions

*YY1* promotes PDAC cell proliferation by enhancing nucleotide availability in a mitochondrial OXPHOS-dependent manner. These findings provide novel therapeutic targets for pancreatic cancer.

## Data Availability

All data and materials are available within the article or from the authors upon reasonable request.
